# Viruses, bacteria, and parasites – oh my! a resurgence of interest in microbial-based therapy for cancer

**DOI:** 10.1186/s40425-017-0312-8

**Published:** 2018-01-08

**Authors:** Andrew Zloza

**Affiliations:** 1 Section of Surgical Oncology Research, Division of Surgical Oncology, Rutgers Cancer Institute of New Jersey, New Brunswick, NJ USA; 2 Department of Surgery, Rutgers Robert Wood Johnson Medical School, Rutgers, The State University of New Jersey, New Brunswick, NJ USA

**Keywords:** Microbial-based cancer treatment, Oncogenic virus, Oncolytic virus, Pathogen, Immunotherapy

## Abstract

As infections and cancer are two of the most common maladies affecting human beings, a concerted effort is needed to better understand their potential interactions and to further explore their use in microbial-based cancer treatments. Studies focusing on the interaction between pathogens and cancer began over 4000 years ago, but therapeutic application of pathogens has often been bypassed as other cancer therapies have gained wider interest. To many, the field of microbial-based cancer treatment may feel antiquated and already sufficiently explored. However, closer examination reveals that our current knowledge is but a series of dim reflections amongst many yet-unexplored shadows. Particularly, with our increased understanding of pathogen entry, replication, and senescence, coupled with our quickly increasing knowledge regarding cancer initiation, growth, and metastasis, and capped by our realization of the complexity and plasticity of the immune response, we are just now beginning to realize the vastness of the undiscovered area encompassing this field. At the same time, we are now uniquely poised with gained knowledge and discovered tools to join together across disciplines, uncover new positive and negative interactions between pathogens and cancer, and make important progress toward saving cancer patient lives.

## Commentary

*Suppose [an] imaginary [scientist] is shown an experiment in which a virus particle enters a [cancer] cell and 20 min later the cell is lysed and 100 virus particles are liberated. [The scientist] will say: “That is very interesting. Let us find out how it happens! How does the particle get in to the [cell]? How does it multiply? Does it have to be inside the [cell] to do this multiplying? This is so simple a phenomenon that the answers cannot be hard to find. In a few months we will know. All we have to do is to study how conditions will influence the multiplication. We will do a few experiments at different temperatures, in different media, with different viruses, and we will know. Perhaps we may have to break into the [cell] at intermediate stages between infection and lysis. Anyhow, the experiments only take a few hours each, so the whole problem cannot take long to solve.”*

[Eight years later] he has not got[ten] anywhere in solving the problem he set out to solve ….

*Adapted from ‘Experiments with Bacterial Viruses (Bacteriophages)’, Harvey Lecture (1946), 41, 161–162.*

It has been more 2 years since the first oncolytic virus in the United States was approved by the U.S. Food and Drug Administration for treatment of cancer and more than 50 years since the first select pathogens were demonstrated to have oncogenic potential. It has been more than 100 years since bacterial toxins (administered into tumors by William Coley and others) were shown to reduce some cancers and more than 4000 years since Egyptian physician Imhotep noted tumor regression after infection was produced by incisions made into tumors (reviewed in [1–3]). Yet, although many significant findings have been reported during bursts of interest in this field [4–9], we remain extraordinarily ignorant regarding the depth of the interactions between pathogens and cancer, and further, how to harness these interactions as a treatment for cancer.

In recent times, interest in the field of microbial-based therapies has expanded and contracted as the effects of pathogens on cancer outcomes (positive versus negative) have made several mid-course reversals. This has included 1) increased interest with reports of the successes surrounding Coley’s toxins, 2) followed by diversion as a select group of pathogens was reported to be oncogenic (directly by integrating in and altering the genome of a previously normal cell or indirectly by causing sustained inflammation), 3) proceeded by recent reinvigoration based on discoveries that select pathogens can be oncolytic (directly infecting cancer cells through utility of pathogen-immune-avoidance mechanisms and through the resultant improvement in tumor microenvironment-based immunity), and 4) culminating in the approval of several viruses as the first oncolytic pathogen therapies for cancer [1]. Similarly on a smaller scale, the effort in my lab has transitioned from our discovery of mechanisms underlying negative effects of non-oncogenic, non-oncolytic pathogens on cancer outcomes to the positive effects of these same pathogens under different conditions, and then full circle again ([4] and personal observations). The most important thing that we have realized in this effort is that a wide array of variables dictates the ultimate effect of host infection on cancer growth. Amongst these are the 1) timing of infection in relation to the progression of the tumor (i.e., prior to clinical appearance versus after tissue establishment of a primary tumor), 2) level of distribution within the host (primary alone versus primary with metastasis), 3) tumor location in reference to the infection (local versus distant), 4) the overlap in sequence between the pathogen and tumor, and 5) the difference in immune response generated by different families of pathogens (i.e., the distribution and timing of cytokines and chemokines), etc. Further, making the field even more complicated, these variables work in concert thus creating an ever-growing (although ultimately, finite and comprehendible) matrix.

Although much is left to be uncovered, the fact that a proportion of patients treated with such therapy experiences long-term benefit is the proof-of-concept that this type of treatment can indeed be harnessed. Therefore, it is imperative that a new field rises to meet the next set of challenges in cancer treatment as traditional cancer therapies are augmented or replaced by cancer immunotherapy, and as cancer immunotherapies progress from investigative trials to commonly used evidence-based combinations, and we look beyond current treatments to the next great advance. Based on the basic tenant of immunology, this line of study may revolutionize the strength and breadth of response that we can inherently produce against cancer. Specifically, anti-cancer (i.e., altered-self) responses are inherently weak because major effectors are deleted in large part by central tolerance and minor (cross-reactive) effectors (that survive positive selection) are muted by peripheral tolerance, all in an effort to protect the host from self destruction [i.e., autoimmunity]). However, anti-pathogen (i.e., non-self, foreign) responses are inherently strong (towards protecting the host against a myriad of pathogens that can at times lead to rapid death). Harnessing anti-pathogen responses against cancer may lead to a “physiologic cure” (i.e., complete elimination of the cancer) or “functional cure” (i.e., maintenance of immunological control long-term until death from unrelated causes).

Importantly, the National Cancer Institute (NCI) has recently made a strong effort to aid the resurgence of interest in this field by facilitating the “NCI Conference on Microbial-based Cancer Therapy.” On July 11–12, 2017, scientists from academia, industry, and the government gathered at the Natcher Center on the Bethesda NIH campus to “describe the complex nature of the microbe-tumor interaction and discuss recent advances in the field … to present current research and to stimulate new research to harness the unique potential of viruses and bacteria to invade, damage or destroy human cells and induce immune responses to create new safe and effective therapeutic approaches to selectively eliminate cancer cells. [10]” The meeting highlighted “opportunities for microbial based therapy where conventional therapy is inadequate such as tumor cell dormancy, tumor cells that are not well accessed by drugs, hypoxia or poorly vascularized tumors. [10]” The meeting was initiated by the NCI Office of Cancer Complementary and Alternative Medicine and supported by the Divisions of Cancer Biology, Cancer Treatment and Diagnosis, Cancer Prevention and the NCI Small Business Innovation Research program to “stimulate more research interest in the field and unleash new tools based on bacteria and viruses against cancer, augmenting NCI's efforts to find novel approaches to combat cancer” [10].

To further advance the field of microbial-based cancer treatments a concerted effort is needed from those with expertise in infectious diseases (towards discovering and identifying the best pathogens to utilize), cancer biology (to understand the mechanisms by which pathogens and anti-pathogen immune responses alter inherent tumor characteristics), immunology (to merge infectious disease and cancer biology knowledge in the context of immune response initiation, differentiation, memory formation, tissue-residence, and recall), and clinical medicine (to progress the basic and translational findings made by scientists to patient treatments). Further efforts are needed likewise by molecular biologists (to compare overlapping genomes), computational biologists (to parse the wealth of genetic and molecular information generated in studies co-involving two major mediators [cancer and pathogen]), epidemiologists (to inform laboratory researchers and clinicians regarding the natural interactions between pathogens and cancer), and an increasing number of other disciplines.

Towards this, the first step is the education of researchers and clinicians that evidence exists for a positive outcome from harnessing of pathogens for cancer treatment, a realization by all that relatively little is known and much is left to be discovered, and a willingness by investigators to take the uphill road less (and thus far, insufficiently) traveled. Government agencies and industry partners need to continue increasing focus and funding for growth of this field. Recently, good effort has been made in this direction, but again, the above lecture best summarizes the still-existent need for additional efforts and a call to action to work together:

*… [Eight years later] he has not got[ten] anywhere in solving the problem he set out to solve.*

*But [he may say to you] “… I could not do it in a few months. Perhaps it will take a few [years or decades], and perhaps it will take the help of a few dozen other people. But listen to what I have found, perhaps you will be interested to join me.”*

*Adapted from ‘Experiments with Bacterial Viruses (Bacteriophages)’, Harvey Lecture (1946), 41, 161–162.*

## References

[1] Newman JH, Zloza A. Infection: a cause of and cure for cancer. Curr Pharmacol Rep. 2017; doi: 10.1007/s40495-017-0109-y. 10.1007/s40495-017-0109-y PMC5686242 29201631

[2] Kaufman HL, Kohlhapp FJ, Zloza A. Oncolytic viruses: a new class of immunotherapy drugs. Nat Rev Drug Discov. 2015;14(9):642–62. doi: 10.1038/nrd4663. 10.1038/nrd4663 PMC7097180 26323545

[3] Mesri Enrique A, Feitelson MA, Munger K. Human Viral Oncogenesis: A Cancer Hallmarks Analysis. Cell Host & Microbe. 15(3):266–82. doi: 10.1016/j.chom.2014.02.011. 10.1016/j.chom.2014.02.011 PMC3992243 24629334

[4] Kohlhapp FJ, Huelsmann EJ, Lacek AT, Schenkel JM, Lusciks J, Broucek JR, Goldufsky JW, Hughes T, Zayas JP, Dolubizno H, Sowell RT, Kuhner R, Burd S, Kubasiak JC, Nabatiyan A, Marshall S, Bommareddy PK, Li SG, Newman JH, Monken CE, Shafikhani SH, Marzo AL, Guevara-Patino JA, Lasfar A, Thomas PG, Lattime EC, Kaufman HL, Zloza A. Non-oncogenic acute viral infections disrupt anti-cancer responses and lead to accelerated cancer-specific host death. Cell Rep. 2016;17(4):957–65. doi: 10.1016/j.celrep.2016.09.068. 10.1016/j.celrep.2016.09.068 PMC5589518 27760326

[5] Ribas A, Dummer R, Puzanov I, VanderWalde A, Andtbacka RHI, Michielin O, Olszanski AJ, Malvehy J, Cebon J, Fernandez E, Kirkwood JM, Gajewski TF, Chen L, Gorski KS, Anderson AA, Diede SJ, Lassman ME, Gansert J, Hodi FS, Long GV. Oncolytic Virotherapy promotes Intratumoral T cell infiltration and improves anti-PD-1 immunotherapy. Cell. 2017;170(6):1109–1119 e1110. doi: 10.1016/j.cell.2017.08.027. 10.1016/j.cell.2017.08.027 PMC8034392 28886381

[6] Kohler M, Ruttner B, Cooper S, Hengartner H, Zinkernagel RM (1990). Enhanced tumor susceptibility of immunocompetent mice infected with lymphocytic choriomeningitis virus. Cancer Immunol Immunother.

[7] Coley WB (1891). II. Contribution to the knowledge of sarcoma. Ann Surg.

[8] Iheagwara UK, Beatty PL, Van PT, Ross TM, Minden JS, Finn OJ. Influenza virus infection elicits protective antibodies and T cells specific for host cell antigens also expressed as tumor-associated antigens: a new view of cancer immunosurveillance. Cancer Immunol Res. 2014;2(3):263–73. doi: 10.1158/2326-6066.CIR-13-0125. 10.1158/2326-6066.CIR-13-0125 PMC4006373 24778322

[9] Andtbacka RH, Kaufman HL, Collichio F, Amatruda T, Senzer N, Chesney J, Delman KA, Spitler LE, Puzanov I, Agarwala SS, Milhem M, Cranmer L, Curti B, Lewis K, Ross M, Guthrie T, Linette GP, Daniels GA, Harrington K, Middleton MR, Miller WH, Jr., Zager JS, Ye Y, Yao B, Li A, Doleman S, VanderWalde A, Gansert J, Coffin RS Talimogene Laherparepvec improves durable response rate in patients with advanced melanoma. J Clin Oncol (2015) 33 (25):2780–2788. doi:10.1200/JCO.2014.58.3377. 10.1200/JCO.2014.58.3377 26014293

[10] White JD. https://ncifrederick.cancer.gov/events/PointOfCare2017/default.asp.

